# IT-Related Barriers and Facilitators to the Implementation of a New European eHealth Solution, the Digital Survivorship Passport (SurPass Version 2.0): Semistructured Digital Survey

**DOI:** 10.2196/49910

**Published:** 2024-05-02

**Authors:** Ismay A E de Beijer, Selina R van den Oever, Eliana Charalambous, Giorgio Cangioli, Julia Balaguer, Edit Bardi, Marie Alfes, Adela Cañete Nieto, Marisa Correcher, Tiago Pinto da Costa, Alexander Degelsegger-Márquez, Vanessa Düster, Anna-Liesa Filbert, Desiree Grabow, Gerald Gredinger, Hannah Gsell, Riccardo Haupt, Maria van Helvoirt, Ruth Ladenstein, Thorsten Langer, Anja Laschkolnig, Monica Muraca, Saskia M F Pluijm, Jelena Rascon, Günter Schreier, Zuzana Tomášikova, Florian Trauner, Justas Trinkūnas, Kathrin Trunner, Anne Uyttebroeck, Leontien C M Kremer, Helena J H van der Pal, Catherine Chronaki, PanCareSurPass Consortium

**Affiliations:** 1 Princess Máxima Center for Pediatric Oncology Utrecht Netherlands; 2 Health Level Seven Europe Brussels Belgium; 3 Venizeleio General Hospital of Heraklion Heraklion Greece; 4 Hospital Universitario y Politécnico La Fe Valencia Spain; 5 St Anna Children’s Hospital Vienna Austria; 6 Department of Paediatrics and Adolescent Medicine Kepler University Hospital Johannes Kepler University Linz Linz Austria; 7 Childhood Cancer International Europe Vienna Austria; 8 Instituto Investigación Sanitaria La Fe Valencia Spain; 9 Gesundheit Österreich GmbH Vienna Austria; 10 Department of Studies and Statistics for Integrated Research and Projects St Anna Children’s Cancer Research Institute Vienna Austria; 11 Division of Childhood Cancer Epidemiology, German Childhood Cancer Registry Institute of Medical Biostatistics, Epidemiology and Informatics University Medical Center of the Johannes Gutenberg University Mainz Mainz Germany; 12 Diagnosi, Osservazione, Prevenzione dopo trattamento Oncologico Clinic Department of Hematology/Oncology Istituto di Ricovero e Cura a Carattere Scientifico Istituto Giannina Gaslini Genova Italy; 13 University Hospitals Leuven, Katholieke Universiteit Leuven Leuven Belgium; 14 Department of Paediatrics Medical University of Vienna Vienna Austria; 15 Universitätsklinikum Schleswig-Holstein Campus Lübeck Lübeck Germany; 16 Vilnius University Hospital Santaros Klinikos Vilnius Lithuania; 17 Clinics for Children’s Diseases, Institute of Clinical Medicine Faculty of Medicine Vilnius University Vilnius Lithuania; 18 Center for Health and Bioresources Austrian Institute of Technology Graz Austria; 19 Wilhelmina Children’s Hospital University Medical Center Utrecht Utrecht Netherlands; 20 Emma Children’s Hospital Amsterdam UMC University of Amsterdam Amsterdam Netherlands; 21 See Acknowledgments

**Keywords:** pediatric oncology, long-term follow up care, survivorship, cancer survivors, Survivorship Passport, SurPass, eHealth, information and technology

## Abstract

**Background:**

To overcome knowledge gaps and optimize long-term follow-up (LTFU) care for childhood cancer survivors, the concept of the Survivorship Passport (SurPass) has been invented. Within the European PanCareSurPass project, the semiautomated and interoperable SurPass (version 2.0) will be optimized, implemented, and evaluated at 6 LTFU care centers representing 6 European countries and 3 distinct health system scenarios: (1) national electronic health information systems (EHISs) in Austria and Lithuania, (2) regional or local EHISs in Italy and Spain, and (3) cancer registries or hospital-based EHISs in Belgium and Germany.

**Objective:**

We aimed to identify and describe barriers and facilitators for SurPass (version 2.0) implementation concerning semiautomation of data input, interoperability, data protection, privacy, and cybersecurity.

**Methods:**

IT specialists from the 6 LTFU care centers participated in a semistructured digital survey focusing on IT-related barriers and facilitators to SurPass (version 2.0) implementation. We used the fit-viability model to assess the compatibility and feasibility of integrating SurPass into existing EHISs.

**Results:**

In total, 13/20 (65%) invited IT specialists participated. The main barriers and facilitators in all 3 health system scenarios related to semiautomated data input and interoperability included unaligned EHIS infrastructure and the use of interoperability frameworks and international coding systems. The main barriers and facilitators related to data protection or privacy and cybersecurity included pseudonymization of personal health data and data retention. According to the fit-viability model, the first health system scenario provides the best fit for SurPass implementation, followed by the second and third scenarios.

**Conclusions:**

This study provides essential insights into the information and IT-related influencing factors that need to be considered when implementing the SurPass (version 2.0) in clinical practice. We recommend the adoption of Health Level Seven Fast Healthcare Interoperability Resources and data security measures such as encryption, pseudonymization, and multifactor authentication to protect personal health data where applicable. In sum, this study offers practical insights into integrating digital health solutions into existing EHISs.

## Introduction

More and more children and adolescents successfully survive cancer into adulthood due to improvements in childhood cancer treatment [1,2]. There are currently around 500,000 childhood cancer survivors in Europe [1-3], with around 8000-10,000 new survivors each year [4]. However, despite the increasing survival rates, childhood cancer survivors remain at risk of impaired quality of life and extensive morbidity and mortality due to disease relapse or late health complications caused by cancer treatments (late effects) [5-7]. Late effects can include physical as well as psychological and social conditions, ranging from subsequent neoplasms and cardiotoxicity to chronic pain and poor psychological well-being [4-16]. To improve or preserve the quality of life of childhood cancer survivors, long-term follow-up (LTFU) care focusing on late-effects surveillance and timely intervention is essential [17]. However, comprehensive LTFU programs are lacking in many pediatric cancer centers [18]. In particular, the coordination of LTFU care between health care providers (HCPs), care managers, and childhood cancer survivors, as well as the available knowledge about late effects and the transition from pediatric to adult health care services, often call for improvement [18-20].

Previous studies have highlighted the need for a treatment summary and care plan for childhood cancer survivors as part of successful LTFU care [21-23]. To increase knowledge among HCPs and childhood cancer survivors and optimize long-term survivorship care, the Survivorship Passport (SurPass) was developed by the Pan-European Network for Care of Survivors after Childhood and Adolescent Cancer (PanCare), a multidisciplinary and international association of professionals, childhood cancer survivors, and their families, aiming to reduce the impact of late health effects for childhood cancer survivors [24-26]. The SurPass summarizes cancer and treatment-related data of childhood cancer survivors and, thanks to built-in algorithms, suggests personalized follow-up recommendations based on evidence-based surveillance guidelines developed by the International Late Effects of Childhood Cancer Guideline Harmonization Group and consensus-based recommendations formulated within several PanCare projects (PanCareSurFup and PanCareFollowUp) [17,26]. In addition, the SurPass provides plain language information on late effects and self-care. All in all, the SurPass supports personalized follow-up care and can improve understanding of late effects among HCPs and childhood cancer survivors. Over the years, multiple versions of SurPass have been developed. At the Istituto Giannina Gaslini, Italy, the SurPass (version 1.2) was found to have an overall positive impact on survivors and their families [24]. Ultimately, the SurPass has the potential to be used throughout Europe and beyond to improve LTFU care and empower childhood cancer survivors to take charge of their own health [24].

Previously developed versions of the SurPass require manual entry of individual treatment data to be entered manually into the SurPass database, making its use in daily clinical practice rather time-consuming [24]. As a result, SurPass is currently being upgraded to a semiautomated and interoperable version (SurPass version 2.0) as part of the European Horizon 2020-funded PanCareSurPass (PCSP) project [27]. Like previous versions of SurPass (version 2.0) will generate a survivor-specific treatment summary and survivorship care plan (SCP) using algorithms that link treatment data with available follow-up recommendations. Unlike previous versions, SurPass (version 2.0; hereafter referred to as SurPass) will facilitate semiautomated data entry from electronic health information systems (EHISs) and integration of SurPass into national or regional electronic health records (EHRs). A high level of interoperability and data protection or security are essential to achieve semiautomated data transfer. The development and harmonization of interfaces is required to enable data exchange between systems and the storage of information in different systems (eg, hospital systems, clinical trials, and cancer registries). Interoperability between EHISs and EHRs and the SurPass platform is facilitated by the Health Level Seven (HL7) Fast Healthcare Interoperability Resources (FHIR) interoperability standard [28]. HL7 FHIR supports the exchange of data between health care software systems and combines the hallmarks of the established HL7 (version 2 and 3) and Clinical Document Architecture (CDA) standards while leveraging current web standards such as XML, JSON, HTTP, and the OAuth (Open Authorization Standard) [29]. In addition, HL7’s international patient summary (IPS) standard specifies an EHR extract containing essential health information intended for use in cross-border care scenarios. IPS models support continuity of care for patients and coordination of care across health systems [30]. Specifically, the treatment summary variables in SurPass are linked according to the IPS. Finally, SurPass has been certified as a Class 1 medical device and must guarantee data security in terms of availability, confidentiality, and data integrity by the General Data Protection Regulation (GDPR) [31] and national data protection and privacy requirements.

Upon successful implementation of SurPass throughout 3 European health system scenarios (national EHISs, regional EHISs, and cancer registries or hospital-based EHISs), the PCSP project must focus on the three main challenges described above: (1) semiautomation of data input, (2) interoperability, and (3) data protection or privacy and cybersecurity. To support the most appropriate implementation strategy for SurPass throughout the 3 health system scenarios and overcome the 3 main challenges, a digital survey study was designed. The results of the first part of the survey, which addressed barriers and facilitators related to the care process and ethical, legal, social, and economic aspects of implementation, are described elsewhere [32]. This report describes the results of the second part of the survey, with which we aimed to identify IT-related barriers and facilitators to SurPass implementation. Subsequently, we aimed to derive insights that could be broadly applied to countries with similar types of health systems interested in implementing SurPass.

## Methods

### Study Design and Participants

The PCSP project representatives were asked to provide the email addresses of all the IT specialists working in their center who are responsible for the management of IT systems used to document the treatment of patients with cancer and the future implementation of SurPass (N=20). Specifically, the IT specialists invited were based in 6 LTFU care centers (hereafter referred to by their country name): 1 IT specialist from Austria (Children’s Cancer Research Institute St Anna Kinderkrebsforschung), 1 from Belgium (Katholieke Universiteit Leuven and University Hospitals Leuven), 5 from Germany (University Medical Center Mainz [German Childhood Cancer Registry] and Universität zu Lübeck), 7 from Italy (Istituto Giannina Gaslini), 2 from Lithuania (Viesoji Istaiga Vilniaus Universiteto Ligonine Santaros Klinikos), and 4 from Spain (Fundación para la Investigación del Hospital Universitario la Fe de la Comunidad Valenciana). The participating centers represented the 3 European health system scenarios, including (1) nationally based EHISs or EHRs (Austria and Lithuania), (2) institutional or regional EHISs or EHRs (Italy and Spain), and (3) national cancer registries and hospital-based EHISs or EHRs (Germany and Belgium).

### Survey Development

The survey was designed using the input from 6 earlier semistructured interviews with IT specialists from each of the participating centers, conducted by researchers from HL7 Europe [33]. The interviews were conducted to build up a picture of the relevant issues to be explored in the survey. In turn, the survey aimed to collect detailed data on the health system scenarios represented by the centers. First, we inquired about individual respondent characteristics, such as country of residence and organization of the IT department, followed by questions about which systems could be accessed by survivorship care staff; whether the information could be downloaded, entered, or updated; whether the information was integrated transparently, via common identifiers, or by other means; and whether the systems could exchange information using application programming interfaces (APIs). Second, respondents were asked to indicate which health data exchange (HDE) standards or interoperability frameworks were used or currently implemented in their institution. Third, the availability of childhood cancer survivor information (eg, medical history, diagnostic imaging, or pathology laboratories) and its format (eg, hardcopy, PDF, or HL7 CDA) and accessibility were examined. Similar questions about availability and format were asked about cancer diagnosis and treatment (eg, histology-cytology reports, cumulative doses of chemotherapy or radiotherapy, and types of immunotherapy) and noncancer medical information (eg, comorbidities, surgical procedures, and hereditary syndromes). Lastly, respondents were asked about data protection, data storage, and available resources related to the implementation of SurPass. The survey concluded with open-ended questions about barriers and facilitators to the implementation of the interoperable SurPass. The full survey is presented in Multimedia Appendix 1.

### Ethical Considerations

Ethical review board or institutional approvals were required and obtained in Belgium (Ethics Committee Research UZ Leuven; S65576), Italy (Comitato Etico Regionale della Liguria; 385/2021, databank ID 11633), Germany (Ethik-Komission Universität zu Lübeck; 21-257), and Spain (Comité de Ética de la Investigación con medicamentos; 2019-170-1). Ethical approval was not required in Austria and Lithuania. Institutional approval for pseudonymized data collection, analysis, and storage was granted by the Princess Máxima Center, the Netherlands (Clinical Research Committee Princess Máxima Center). This study was performed in line with the principles of the Declaration of Helsinki. Written informed consent was obtained from all participants included in this study. All responses to the survey were pseudonymized. Participants were informed that by participating in this study, they consented to the publication of their pseudonymized responses.

### Data Collection

Survey distribution and data collection were conducted using the cloud-based central data management platform Castor electronic data capture, a GDPR-compliant digital survey tool. The survey was conducted in English. On-site language assistance was provided to participants with limited English proficiency. The survey was launched on August 16, 2021, and closed on October 4, 2021. The results were sent to the centers for validation, which took place from December 2021 to March 2022.

### Data Analysis

Pseudonymized survey data were exported from Castor electronic data capture and processed and analyzed using SPSS (version 25.0, IBM Corp). The survey results were categorized and analyzed per health system scenario and according to the three main challenges: (1) semiautomation of data input, (2) interoperability, and (3) data protection or privacy and cybersecurity. Additionally, the availability of sufficient resources (ie, staff, funding, and knowledge) to implement SurPass was considered separately for each center.

Predefined barriers to semiautomated data input and interoperability included: unaligned EHIS infrastructures without the availability of a personalized EHR or structured centralized clinical trial database from which (clinical) data could be transferred; the availability or accessibility of less than half of the requested health data information sources and patient data; the nonuse of HL7 FHIR; and the use of proprietary (noninteroperable) coding systems. Barriers to data protection or privacy and cybersecurity included not being able to privacy-protect SurPass record linkage (in line with GDPR), less than half of the IT specialists (per center) being able to guarantee data protection, and less than half of the IT specialists (per center) being familiar with data retention regulations. Finally, the unavailability of sufficient resources as indicated by at least half of the IT specialists (per center), was also considered a barrier.

Predefined facilitators for semiautomation of data input and interoperability included having a standardized and structured HDE process; the availability of a personalized EHR or structured centralized clinical trial database from which (clinical) data could be transferred in the case of nonaligned EHISs; the availability or accessibility of at least half of the requested health data information sources and patient data, ideally with the possibility to download, enter, update, and integrate the data using APIs; the use of HL7 FHIR; and the use of international coding systems such as the Anatomical Therapeutic Chemical (ATC) code system, the *International Classification of Diseases for Oncology, 3rd Edition* (*ICD-O-3*), and the *International Classification for Childhood Cancer, 3rd Edition* (*ICCC-3*) as used in SurPass. Facilitators for data protection or privacy and cybersecurity included pseudonymization of personal health data per the European Regulation 2016/679 of the European Parliament and of the Council of April 27, 2016, on the protection of natural persons per the processing of personal data and the free movement of such data, at least half of the IT specialists (per center) being able to ensure data protection, and at least half of the IT specialists (per center) being familiar with data retention regulations. Similarly, the availability of sufficient resources, as indicated by at least half of the IT specialists (per center), was considered to facilitate the implementation of SurPass.

Furthermore, we used the fit-viability model (FVM) as described by Liang et al [34] to assess the successful implementation of SurPass across the 3 unique health system scenarios. Specifically, the FVM evaluates the technological factors and organizational readiness essential for implementing information technology. Liang et al [34] defined “fit” as the match between task requirements and technology capabilities and “viability” as the economic feasibility, IT infrastructure maturity, and organizational support. In our study, we used the FVM to assess how well SurPass fits into the 3 health system scenarios and the extent to which SurPass is feasible, sustainable, and likely to be successfully integrated into the existing EHIS in the different health system scenarios, taking into account the system-specific barriers and facilitators.

## Results

### Survey Participants

A total of 13 IT specialists (1 from Austria and Belgium, 2 from Lithuania and Spain, 3 from Germany, and 4 from Italy) responded to the survey invitation. Further, 3 IT specialists represented the first scenario (national EHISs), 5 IT specialists represented the second scenario (regional or local EHISs), and 4 IT specialists represented the third scenario (cancer registries and hospital-based EHISs). The overall response rate was 65%. The response rates per country were 100% for Austria (1/1), Belgium (1/1), and Lithuania (2/2); 60% (3/5) for Germany; 57% (4/7) for Italy, and 50% (2/4) for Spain.

### Barriers and Facilitators per Health System Scenario

#### Overview of Available Data Systems and Resources

Inherent to the 3 health system scenarios, there are barriers and facilitators relevant to the implementation of SurPass. The characteristics of these scenarios, categorized according to the 3 main implementation challenges, are described below. The availability of data systems and resources in each center is discussed accordingly (see also Tables 1-4). The center-specific HDE standards, interoperability frameworks, and coding systems for each center are described separately by Chronaki et al [33]. A general overview of all the identified barriers and facilitators to the implementation of the SurPass is provided in Table 5.

**Table 1:** Information system sources in each health system scenario and center.

Accessible data systems		Scenario 1			Scenario 2			Scenario 3	
		Lithuania	Austria	Italy		Spain	Belgium		Germany
**National EHR^a^**		Yes	Yes	Yes		Yes	Yes		No
	Possibility to download data	Yes	Yes	Yes		Yes	Yes		No
	Possibility to enter data	Yes	*m^b^*	Yes		No	Yes		No
	Possibility to update data	Yes	Yes	Yes		No	No		No
	Integrated transparently	No	*m*	Yes		No	No		No
	APIs^c^ available to integrate	Yes	Yes	Yes		No	Yes		No
**Regional EHR**		?^d^	*m*	~^e^		Yes	No		No
	Possibility to download data	?	*m*	Yes		No	No		No
	Possibility to enter data	?	*m*	Yes		Yes	No		No
	Possibility to update data	?	*m*	Yes		Yes	No		No
	Integrated transparently	?	*m*	?		~	No		No
	APIs available to integrate	?	*m*	Yes		~	No		No
**Cancer registry**		Yes	Yes	Yes		Yes	Yes		Yes
	Possibility to download data	No	Yes	Yes		?	Yes		No
	Possibility to enter data	No	Yes	Yes		Yes	Yes		Yes
	Possibility to update data	No	Yes	Yes		Yes	No		Yes
	Integrated transparently	?	*m*	?		Yes	No		?
	APIs available to integrate	No	Yes	?		No	Yes		No
**Hospital EMR^f^**		Yes	*m*	Yes		Yes	Yes		Yes
	Possibility to download data	Yes	*m*	Yes		Yes	Yes		No
	Possibility to enter data	Yes	*m*	Yes		Yes	Yes		?
	Possibility to update data	Yes	*m*	Yes		Yes	Yes		?
	Integrated transparently	Yes	*m*	Yes		Yes	Yes		?
	APIs available to integrate	Yes	*m*	Yes		Yes	No		?
**Patient records at LTFU^g^ care center**		Yes	*m*	Yes		Yes	Yes		?
	Possibility to download data	Yes	*m*	Yes		Yes	Yes		?
	Possibility to enter data	Yes	*m*	Yes		Yes	Yes		?
	Possibility to update data	Yes	*m*	Yes		Yes	Yes		?
	Integrated transparently	*m*	*m*	Yes		No	Yes		?
	APIs available to integrate	*m*	*m*	Yes		No	No		?
**Patient records at other outpatient center**		Yes	*m*	No		No	Yes		?
	Possibility to download data	Yes	*m*	No		No	Yes		?
	Possibility to enter data	Yes	*m*	No		No	No		?
	Possibility to update data	Yes	*m*	No		No	No		?
	Integrated transparently	Yes	*m*	No		No	Yes		?
	APIs available to integrate	Yes	*m*	No		No	No		?
**Appointment scheduling system**		Yes	Yes	Yes		Yes	Yes		Yes
	Possibility to download data	Yes	*m*	Yes		Yes	Yes		?
	Possibility to enter data	Yes	Yes	Yes		Yes	Yes		?
	Possibility to update data	Yes	Yes	Yes		Yes	Yes		?
	Integrated transparently	?	*m*	Yes		Yes	Yes		?
	APIs available to integrate	Yes	*m*	Yes		Yes	No		?
**Pharmacy**		Yes	*m*	Yes		Yes	Yes		Yes
	Possibility to download data	?	*m*	Yes		Yes	Yes		?
	Possibility to enter data	?	*m*	Yes		Yes	Yes		?
	Possibility to update data	?	*m*	Yes		Yes	Yes		?
	Integrated transparently	Yes	*m*	Yes		Yes	Yes		?
	APIs available to integrate	?	*m*	Yes		Yes	Yes		?
**Laboratories**		Yes	Yes	Yes		Yes	Yes		Yes
	Possibility to download data	Yes	Yes	Yes		Yes	Yes		?
	Possibility to enter data	No	No	Yes		Yes	Yes		?
	Possibility to update data	No	No	Yes		Yes	Yes		?
	Integrated transparently	?	*m*	Yes		Yes	Yes		?
	APIs available to integrate	?	*m*	Yes		Yes	Yes		?
**Radiology**		Yes	Yes	Yes		Yes	Yes		?
	Possibility to download data	Yes	Yes	Yes		Yes	Yes		?
	Possibility to enter data	Yes	No	Yes		Yes	Yes		?
	Possibility to update data	Yes	No	Yes		Yes	Yes		?
	Integrated transparently	?	*m*	Yes		Yes	Yes		?
	APIs available to integrate	?	*m*	Yes		Yes	Yes		?
**Clinical trial systems**		Yes	*m*	No		No	Yes		Yes
	Possibility to download data	Yes	*m*	No		No	No		?
	Possibility to enter data	Yes	*m*	No		No	No		?
	Possibility to update data	Yes	*m*	No		No	No		?
	Integrated transparently	Yes	*m*	No		No	Yes		?
	APIs available to integrate	Yes	*m*	No		No	No		?
**Primary health care information system**		Yes	Yes	No		Yes	No		Yes
	Possibility to download data	*m*	Yes	No		Yes	No		No
	Possibility to enter data	*m*	Yes	No		Yes	No		?
	Possibility to update data	*m*	Yes	No		Yes	No		?
	Integrated transparently	*m*	*m*	No		Yes	No		?
	APIs available to integrate	*m*	*m*	No		Yes	No		?

^a^EHR: electronic health record.

^b^*m*: Answers missing.

^c^API: application programming interface.

^d^?: Respondents did not know.

^e^~: Unclear, for example, 1 × “yes” and 1 × “no.”

^f^EMR: electronic medical record.

^g^LTFU: long-term follow-up.

**Table 2:** Patient data that is electronically available in each health system scenario and center^a^.

Characteristics		Scenario 1	Scenario 2		Scenario 3	
		Lithuania	Italy	Spain	Belgium	Germany
**Patient data type**						
	Patient summary or medical history	Yes	Yes	Yes	Yes	Yes
	Hospital admissions	Yes	Yes	Yes	Yes	Yes
	Diagnostic imaging (images)	Yes	Yes	Yes	Yes	Yes
	Diagnostic imaging (reports)	Yes	Yes	Yes	Yes	Yes
	Biochemical laboratories	Yes	Yes	Yes	Yes	Yes
	Pathology laboratories	Yes	Yes	Yes	Yes	Yes
	CC^b^ treatment summary	Yes	Yes	Yes	?^c^	Yes
	LTFU^d^ care visit report	?	Yes	No	?	?
	SurPass^e^	No	Yes	No	No	No
	LTFU center appointments	Yes	?	?	?	?
	Other medical databases	?	?	No	Yes	Yes

^a^An earlier version of the Survivorship Passport has been implemented and evaluated previously in Italy. Austria was intentionally left out of the table because all answers were missing.

^b^CC: childhood cancer.

^c^Respondents did not know.

^d^LTFU: long-term follow-up.

^e^SurPass: Survivorship Passport.

**Table 3:** Overview of accessible patient data formats in each health system scenario and center. Austria was intentionally left out of the table because all answers were missing.

Patient data type			Scenario 1		Scenario 2				Scenario 3		
			Lithuania		Italy		Spain		Belgium		Germany
**General medical history**											
	Comorbidities	****^a^		****		?^b^		?		***^c^	
	Allergies	****		****		****		***		****	
	Medication (noncancer related)	****		****		****		***		**^d^	
	Surgical procedures (noncancer related)	****		****		****		***		**	
	Admissions	***		****		***		**		***	
	Trauma	****		****		**		**		No	
	Hereditary syndromes	***		No		**		***		No	
**Cancer diagnosis**											
	Cancer diagnosis	****		?		***		****		**	
	Cancer diagnosis date	****		*^e^		***		^f^		****	
	Histology-cytology report	***		***		****		**		***	
	Imaging reports	**		***		****		**		**	
	Laboratory reports	****		****		***		**		**	
**Cancer treatment**											
	Surgical interventions	**		****		****		**		**	
	Stem cell or bone marrow transplantations	***		**		?		**		No	
	Chemotherapy start or end date	?		**		***		***		**	
	Chemotherapy type	?		***		***		****		**	
	Chemotherapy cumulative dose	**		***		***		?		?	
	Chemotherapy treatment complications	?		**		***		***		?	
	Immunotherapy start or end date	?		?		?		**		?	
	Immunotherapy type	?		?		?		**		?	
	Immunotherapy cumulative dose	?		?		?		?		?	
	Immunotherapy treatment complications	?		?		?		?		?	
	Hormonal therapy start or end date	?		?		?		**		?	
	Hormonal therapy type	?		?		?		**		?	
	Hormonal therapy cumulative dose	?		?		?		?		?	
	Hormonal therapy treatment complications	?		?		?		?		?	
	Radiotherapy start or end date	**		*		***		**		?	
	Radiotherapy type	?		*		***		**		?	
	Radiotherapy cumulative dose	?		*		***		?		?	
	Radiotherapy site	?		*		***		?		?	
	Radiotherapy treatment complications	?		*		***		?		?	

^a^Electronically available, coded, and interconnected.

^b^Respondents did not know.

^c^Yes, electronically available and coded.

^d^Yes, electronically available.

^e^Yes, hardcopy available (awarded when at least one respondent mentioned “yes”).

^f^Electronically available, free text, and interconnected.

**Table 4:** Scarcity of resources in each health system scenario and center. The table shows the number of IT specialists per country that indicated either having (yes) or not having (no) sufficient staff, time, funds, and knowledge.

Resource type lacking, n			Scenario 1				Scenario 2					Scenario 3		
			Lithuania		Austria		Italy		Spain		Belgium			Germany
**Staff**														
	Yes	0		1		1		1		0			2	
	No	2		0		3		1		1				
**Time**														
	Yes	0		1		2		1		0			2	
	No	2		0		2		1		1			1	
**Funds**														
	Yes	0		1		2		1		0			1	
	No	2		0		2		1		1			2	
**Knowledge**														
	Yes	0		1		2		0		0			1	
	No	2		0		2		2		1			2	

**Table 5:** Summary of the identified barriers and facilitators to the implementation of the SurPass^a^ (version 2.0) throughout all 3 health care system scenarios.

Characteristics			Barriers		Facilitators
**Scenario 1: national EHISs^b^**					
	Austria^c^	Uncertainty about data retention Lack of resources		Standardized and structured data exchange Straightforward GDPR^d^ compliance using pseudonymization Data protection can be guaranteed Use of ATC^e^ code system for medication Use of ICD-O-3^f^ for histology	
	Lithuania	Uncertainty about data retention		Standardized and structured data exchange Majority of the data systems is accessible Majority of patient data is available Use of HL7 FHIR^g^ Data protection can be guaranteed Sufficiency of resources Use of ICD-O-3 for histology	
**Scenario 2: regional EHISs**					
	Italy	EHIS infrastructure discrepancies Uncertainty about data protection Uncertainty about data retention Uncertainty about the sufficiency of resources		Availability of personalized EHR^h^ (Fascicolo Sanitario Elettronico) Majority of the data systems is accessible Majority of patient data is available Use of HL7 FHIR Use of ATC code system for medication	
	Spain	EHIS infrastructure discrepancies Unavailability of HDE^i^ standards Uncertainty about data retention Uncertainty about the sufficiency of resources		Availability of personalized EHR (Historia de Salud Electrónica) Majority of the data systems is accessible Majority of patient data is available Data protection can be guaranteed	
**Scenario 3 cancer registries and hospital-based EHISs**					
	Belgium	Data origination from different sources EHIS infrastructure discrepancies Uncertainty about data protection Uncertainty about data retention Uncertainty about the sufficiency of resources		Majority of the data systems is accessible Majority of patient data is available Use of HL7 FHIR Use of ATC code system for medication	
	Germany	Data origination from different sources EHIS infrastructure discrepancies Majority of the data systems is accessible but not downloadable Uncertainty about data protection Uncertainty about data retention Lack of resources		Availability of structured centralized clinical trial databases Majority of patient data is available Use of HL7 FHIR Use of ICD-O-3 and ICCC-3^j^ for cancer diagnosis and histology	

^a^SurPass: Survivorship Passport.

^b^EHIS: electronic health information system.

^c^Accessibility of data systems and patient data availability is unknown due to missing answers from the information technology specialist from Austria.

^d^GDPR: General Data Protection Regulation.

^e^ATC: Anatomical Therapeutic Chemical.

^f^ICD-O-3: International Classification of Diseases for Oncology, 3rd Edition.

^g^HL7 FHIR: Health Level 7 Fast Healthcare Interoperability Resources.

^h^EHR: electronic health record.

^i^HDE: health data exchange.

^j^ICCC-3: International Classification of Childhood Cancer, Third edition.

#### National EHISs (Austria and Lithuania)

In this scenario, the data required for the patient-specific oncological treatment summary will be provided by HCPs through a national EHISs, such as the ELGA (Austrian National Electronic Health Record Systems) or the Lithuanian Electronic Health Services and Infrastructure Cooperation System (ESPBI IS). The patient-specific oncological treatment summary, which takes into account the cumulative treatment burden and extends to the end of treatment of the first pediatric cancer, will be systematically generated in CDA format. This scenario thus provides a standardized and structured way of exchanging data. In addition, the accessibility of digital data is fundamental to enable semiautomated data input from EHISs and to facilitate interoperability using HL7 FHIR. In Lithuania, these conditions are met as all data systems and the majority of patient data are accessible (Tables 1-3) and HL7 FHIR is used. In addition, the IT specialists from Lithuania indicated that they have sufficient resources (staff, funds, and knowledge) available to implement SurPass (version 2.0) in their center (Table 4). On the other hand, the IT specialist from Austria indicated that he did not have sufficient resources at hand. In addition, no information was available on the accessibility of the Austrian data system. In terms of interoperability, Austria currently uses HL7 CDA in ELGA and is working toward the adoption of HL7 FHIR in the future. For medication, Austria uses the ATC code system. For cancer diagnosis, Lithuania uses the *ICCC-3*. For histology, both Lithuania and Austria use the *ICD-O-3*. Other coding systems varied substantially between the 2 centers.

Per privacy and security, the structured data in this scenario will be pseudonymized where necessary, for example by using the European Patient Identity Service (EUPID; pseudonymization and privacy-preserving record linkage tool to facilitate secondary use of data sets in biomedical research and health care [35,36]). Next, the data will be transferred to the SurPass platform to generate the SurPass. EUPID will certainly be used in Austria, but there is no consensus yet in Lithuania. In the future, the use of EUPID for the HDE process will ensure GDPR compliance. In this study, survey results indicated that IT professionals in Austria and Lithuania have existing solutions that ensure SurPass data protection. Once the pseudonymized SurPass is generated, either the entire SurPass (in the case of Lithuania’s Electronic Health Services and Infrastructure Cooperation System) or the SCP (in the case of Austria’s ELGA) will be returned to the HCP and the childhood cancer survivor’s local care team, where the SCP within the SurPass can be tailored to the childhood cancer survivor’s individual needs and national care pathways in the local health IT system. The SurPass will be accessible to both childhood cancer survivors and HCPs through the national EHIS or EHR. However, the IT specialists from Austria and Lithuania were uncertain about SurPass data retention. In the Austrian ELGA, data retention is currently limited to 10 years, which is likely to be too short for the SurPass concept.

In terms of the FVM, this scenario shows a high fit for SurPass, particularly in Lithuania given their current use of HL7 FHIR and the accessibility of data systems. In Austria, where a transition to HL7 FHIR is underway, SurPass compatibility is growing. Viability is strong with evident economic feasibility for Lithuania and effective data protection strategies present in both countries. Thus, prospects for SurPass adoption in this scenario are promising, especially in Lithuania, where ample resources contribute to a favorable implementation environment. Provided that uncertainties about data retention are resolved and Austria allocates sufficient resources, the implementation of SurPass is likely to be successful.

#### Regional EHISs (Italy and Spain)

In the second scenario, the main pediatric cancer institutions in each region have local registries or treatment protocol databases with demographic information and treatment data. Here, EHIS infrastructure is not fully aligned between regions or local hospitals in the same region, limiting semiautomated data exchange and interoperability. Similarly, EHISs may have been adopted at different times and therefore differ in terms of completeness or level of sophistication of the system, limiting interoperability. However, SurPass implementation can be aided by the availability of personalized EHRs (Fascicolo Sanitario Elettronico in Italy and Historia de Salud Electrónica in Spain), which contains medical information that is accessible to all HCPs in the region or country and which also allows patients to access their data. However, it is worth noting that the FSE in Italy is currently undergoing a significant reorganization, transitioning from a regional to a national homogeneous system that will use HL7 FHIR. This transition may lead to changes in the way medical information is accessed and exchanged between HCPs, but it will allow SurPass to be integrated into the Italian EHR. Furthermore, the survey results showed that in both Italy and Spain, the majority of data systems and patient data are accessible to survivorship care staff (Tables 1-3). Apart from the cancer registry, all data systems in Italy are also transparently integrated, including APIs, supporting interoperability between these systems together with the use of HL7 FHIR. Spain, on the other hand, has not yet adopted HL7 FHIR. For medication, Italy uses the ATC system. Other coding systems varied substantially between the 2 centers.

In this scenario, the SurPass (version 2.0) is intended to be integrated into the EHIS of the treating institution for follow-up care, as well as into the EHR for use in possible emergency admissions to other hospitals and during the transition from pediatric to adult LTFU care. Similar to the first scenario, the cross-border data transfer could be pseudonymized by using a GDPR-compliant double pseudonymization system such as EUPID. Personalization of the SurPass data would only take place within the treating institution itself, or in other local databases with permission to hold identifying personal data. The results of the survey showed that, in contrast to the Italian respondents, the majority of IT specialists from Spain indicated that they were able to guarantee the protection of SurPass data. Neither center’s IT specialists knew how long SurPass records had to be stored. Besides, IT specialists in both centers were unsure about the availability of sufficient resources to implement SurPass (Table 4).

In the context of the FVM, this scenario shows a moderate fit for SurPass implementation within regional EHIS structures. Viability is impacted by EHIS infrastructures that are not fully aligned between regions and local hospitals, hindering seamless semiautomated data exchange and interoperability. Despite these barriers, the availability of personalized EHRs offers potential support for SurPass integration. Furthermore, viability is dependent on data protection concerns and GDPR compliance. Provided these challenges are addressed and sufficient resources can be secured, the adoption of SurPass in centers representing this scenario appears promising.

#### Cancer Registries and Hospital-Based EHISs (Belgium and Germany)

The third scenario is the least favorable in terms of semiautomated data input, interoperability, and data protection. Unlike the scenarios with nationally or regionally organized health data, this scenario requires the integration of data from a multitude of sources to generate a SurPass. Epidemiological data will need to be obtained from cancer or bone marrow transplant registries such as the German Childhood Cancer Registry, while disease and treatment-specific clinical data will need to be retrieved from hospital databases. In addition, although it is possible to access and transfer clinical data from central clinical trial databases in Germany, this process adds complexity and is not as streamlined as data collection in the other 2 scenarios. Both Belgium and Germany store SurPass data at CINECA, the largest data center in Italy [37]. The resulting SurPass will be returned digitally to the centers in PDF format. Currently, in both Belgium and Germany, most data systems and patient data are accessible, although in Germany without the possibility of downloading or further exploiting the data (Tables 1-3). Yet, if the data systems are already accessible, the possibilities to download, enter, update, and integrate the data can be explored shortly as applicable during the PCSP project. Moreover, both centers in Belgium and Germany are using HL7 FHIR, which improves interoperability between local health data systems. For cancer diagnosis and histology, Germany uses the cancer-specific coding systems ICD-O-3 and ICCC-3, and Belgium uses the ATC code system for medication. Other coding systems varied substantially between the centers. Lastly, at the time of completing the questionnaire, IT specialists from Belgium and Germany were uncertain about guaranteeing data protection, data retention, and the availability of sufficient resources in their centers (Table 4).

In this scenario, the FVM underscores significant barriers to SurPass implementation. SurPass fit is challenged by the need to integrate data from diverse sources. However, viability is reasonable, with accessible data systems and HL7 FHIR adoption improving interoperability. SurPass adoption or implementation supported by accessible data systems is feasible but will depend on overcoming challenges related to data transfer, data protection, and resource availability.

## Discussion

### Principal Findings

Using a digital survey, we assessed IT-related barriers and facilitators to the implementation of the digital SurPass throughout 3 health system scenarios (national EHISs—Austria and Lithuania; regional or local EHISs—Italy and Spain; and cancer registries or hospital-based EHISs—Belgium and Germany). The survey involved 13 IT specialists from the participating centers. The application of the FVM to the 3 distinct health system scenarios provides valuable insights into the feasibility of implementing SurPass in pediatric cancer survivorship care. Scenario 1 (national EHISs) shows a positive outlook for a successful SurPass implementation, especially in Lithuania, where current HL7 FHIR usage and accessible data systems contribute to a high fit and good viability. In Austria, ongoing efforts to transition to HL7 FHIR indicate growing compatibility. Viability is strong in both countries, setting the stage for promising SurPass adoption, provided that uncertainties around data retention in Austria are addressed and sufficient resources are allocated. Scenario 2 (regional or local EHISs) presents a more nuanced picture with a moderate fit. Viability is hampered by misaligned EHIS infrastructures, impacting semiautomated data exchange and interoperability. Despite these barriers, personalized EHRs offer support for SurPass, provided that data protection concerns are dealt with. However, uncertainties among IT professionals regarding data protection, storage, and resource availability suggest that careful consideration is needed in the adoption process. Finally, scenario 3 (cancer registries or hospital-based EHISs) presents significant barriers to good SurPass fit due to the complexity of integrating data from multiple sources. Nevertheless, a reasonable viability, characterized by accessible data systems and HL7 FHIR adoption, suggests successful SurPass implementation in the future, provided that challenges related to data transfer, data protection, and resource availability are effectively handled. These findings highlight the importance of context-specific barriers and facilitators to the implementation of SurPass in the 3 different health care scenarios.

SurPass implementation requires the exchange of medical data between various data sources, especially in the second and third health system scenarios. National EHISs, as in Austria and Lithuania, are best suited for semiautomated data transfer from the EHIS of the treating institution to the SurPass platform, followed by the regional or local EHIS infrastructures, as in the second and third scenarios. Ultimately, semiautomated data input is paramount to making clinical care processes more efficient. Besides the EHIS infrastructure, the use of HL7 FHIR is of major importance for connecting SurPass to EHISs, registries, and national or regional EHRs. Due to its international recognition and adoption following the open API paradigm, FHIR is an ideal choice for promoting interoperability and the standardized exchange of health care data. In addition to the IPS, FHIR’s flexibility, extensibility, and wide adoption within the health care industry support our decision to recommend FHIR. All centers except Spain and Austria are already using HL7 FHIR. Austria is currently using HL7 CDA in ELGA but is also working toward the adoption of HL7 FHIR. Relatedly, a systematic literature analysis on barriers and facilitators for the implementation of eHealth services found that having well-established HDE standards is an important success factor for eHealth services [38]. Furthermore, the lack of time for HCPs to create and update the SurPass is an important barrier in the survivorship care process [32]. Since the time needed to prepare and update SurPass depends on the level of interoperability that can be achieved between existing EHISs and SurPass, we recommend the LTFU care center in Spain and any other institution with the ambition to implement SurPass to adopt HL7 FHIR in all EHISs.

Complications with data accessibility and data privacy or security are listed in the top 10 barriers mentioned in 38 papers on the implementation of eHealth solutions [38]. On the one hand, this study illustrated that the majority of information sources and patient data types is accessible and downloadable in all health system scenarios, except in Germany (scenario 3), where the data are accessible but not downloadable. On the other hand, only in the first scenario (Austria and Lithuania) and in Spain (scenario 2) did IT specialists report being able to ensure SurPass data protection. Per data retention, IT specialists exhibited uncertainty and inconsistency in all scenarios. It is important to consider the context of the survey when interpreting these results. For example, in Germany, IT specialists were responsible for software development but were not involved in detailed data protection discussions, which were handled by the official data protection officer. Each of the IT specialists involved is aware of the necessary prerequisites to ensure data protection. They may need to establish a specific framework to effectively integrate SurPass data and comply with data protection standards. It remains imperative for all centers to have a data protection solution in place, as compliance with GDPR and national requirements for data protection and privacy is an essential prerequisite for the successful implementation of SurPass in all health system scenarios. Data encryption, pseudonymization, and multifactor authentication—a security process in which a user provides two or more authentication factors to verify their identity before accessing a protected resource or system—could form appropriate data protection measures for all health system scenarios.

The insights gained from SurPass implementation have broader applicability, offering valuable lessons for the introduction of similar eHealth solutions in various health care settings. SurPass serves as a practical example illustrating the integration of digital health tools into existing EHISs, and the application of the FVM framework provides essential technological considerations for implementing such solutions beyond the SurPass context. In addition, our study is of strategic importance in light of the upcoming European Health Data Space (EHDS) regulation, which refines the GDPR and requires electronic health systems to be able to exchange data in the European Electronic Health Record Exchange Format [39]. SurPass has many common elements with the IPS, which is one of the priority data categories in the EHDS regulation. In view of this, our study, despite its specific focus, sheds light on some of the hurdles of digital transformation in Europe as it explores the health data economy in realistic care pathways. As such, SurPass lays the groundwork for the pragmatic adoption of key regulations and upcoming directives such as the EHDS, the Medical Device Directive, and the Artificial Intelligence Act [39-41].

### Limitations

The limitations of this study include missing data from Austria regarding accessibility of data systems and availability of patient data, and inconsistent or uncertain responses within individual centers. This may be because SurPass is a newly developed tool and many IT specialists were unfamiliar with SurPass and its requirements. In addition, there were many unknown responses from German centers regarding information system sources, which may be attributed to the complexity of the IT systems in place and the lack of involvement of German IT specialists in detailed discussions on data protection. We, therefore, acknowledge that the overview of computing infrastructures and IT landscapes across the 6 centers may be incomplete. Furthermore, the number of IT specialists participating in this study may appear relatively small. The selection process was based on their direct involvement in the project to ensure a relevant perspective on the SurPass implementation. This study’s design including 1 center per country was based on resource allocation and practical considerations, as outlined in the PCSP project agreement. Despite initially inviting IT specialists from each of the 6 participating centers (N=20), only 13 provided complete responses. This limited participation reflects the challenges of understaffed and less engaged hospital IT departments across Europe. We acknowledge the potential for selection bias, as their individual expertise was not systematically assessed. However, it is important to note that after analyzing the survey results, we conducted a thorough verification of the IT specialists’ responses with all participating centers. While we recognize this study’s limitations, we emphasize that our findings are of substantial value in shaping an effective implementation strategy for SurPass in all 3 health system scenarios. To facilitate the wider adoption of SurPass, we plan to publish an implementation toolkit at the end of the PCSP project.

### Conclusions

This paper described the findings from a digital survey study that assessed IT-related barriers and facilitators to SurPass implementation in 3 European health system scenarios, that is nationally based EHISs or EHRs (Austria and Lithuania); institutional or regional EHISs or EHRs (Italy and Spain); and national cancer registries and hospital-based EHISs or EHRs (Germany and Belgium). In all scenarios, barriers and facilitators were related to 3 main challenges including semiautomated data input, interoperability, and data protection or privacy and cybersecurity. For all scenarios, we recommend the adoption of HL7 FHIR to support interoperability. In addition, we emphasize the importance of GDPR compliance in all scenarios, such as using encryption, EUPID pseudonymization, and multifactor authentication where applicable. Our results will support the SurPass implementation strategies for all 3 health system scenarios. Ultimately, SurPass implementation provides lessons for introducing similar eHealth solutions across diverse health care settings, offering practical insights into integrating digital health solutions into existing and future EHISs and paving the way for the pragmatic adoption of key regulations such as the EHDS, the Medical Device Directive, and the Artificial Intelligence Act.

## Appendix

Multimedia Appendix 1 Full survey.

## Abbreviations

ATC: Anatomical Therapeutic Chemical
CDA: clinical document architecture
EHDS: European Health Data Space
EHIS: electronic health information system
EHR: electronic health record
ELGA: Austrian National Electronic Health Record Systems
EUPID: European Patient Identity Service
FHIR: Fast Healthcare Interoperability Resources
FVM: fit-viability model
GDPR: General Data Protection Regulation
HCP: health care provider
HDE: health data exchange
HL7: Health Level Seven
ICCC-3: International Classification for Childhood Cancer, 3rd Edition
ICD-O-3: International Classification of Diseases for Oncology, 3rd Edition
IPS: international patient summary
LTFU: long-term follow-up
OAuth: Open Authorization Standard
PCSP: PanCareSurPass
SCP: survivorship care plan
SurPass: Survivorship Passport

## Data Availability

Study participants did not consent to data sharing outside the PanCareSurPass project. Access to participant data is therefore limited to national and international supervisory authorities. This study’s protocol can be made available upon request to the corresponding author.
