# Supplementary material for: IT-Related Barriers and Facilitators to the Implementation of a New European eHealth Solution, the Digital Survivorship Passport (SurPass Version 2.0): Semistructured Digital Survey
Source: J Med Internet Res. 2024 May 2;26:e49910. doi: 10.2196/49910 (PMC11099813; doi:10.2196/49910)
Supplement: Multimedia Appendix 1 [file jmir_v26i1e49910_app1.pdf]

Multimedia Appendix 1. Full survey.

Survey IT-specialist

Title: Implementation of the digital Survivorship Passport (SurPass) in survivorship care

Introduction

Thank you for your interest in this study! Please read this information carefully before completing the survey.

The Survivorship Passport (SurPass) is a tool that provides survivors of childhood cancer and their healthcare providers with the information needed for life-long survivorship care. For each survivor, the SurPass provides:

- **a treatment summary** including the cancer diagnosis and cancer-related treatments received.
- **a personalised survivorship care plan** (including past, current and potential future health problems), based on international evidence-based clinical guidelines.
- **plain language information** about possible late effects of childhood cancer and its treatment.

Where previous versions of the SurPass were paper-based and only allowed for manual data input, the newest version of the SurPass (v2.0) is digital and facilitates semi-automatic patient and treatment data retrieval from existing databases such as the electronic medical record (EMR) and/or cancer registry. During the PanCareSurPass project, we aim to improve care and quality of life for survivors by implementing the SurPass v2.0 in 6 European countries.

The purpose of this survey is to identify barriers and facilitators concerning **information and IT** for the implementation of the digital SurPass (v2.0) in the survivorship care unit at your institution. We will ask you about your computing infrastructure, the degree to which it is interconnected, and the type of information recorded: what information about childhood cancer survivors is paper-based, and what is kept electronically? And what information is available for the semi-automatic generation of the digital SurPass (v2.0)? We end with questions on legal and economical IT aspects.

Your answers to this survey will help us improve the SurPass tool and implement it at your institution and other institutions in Europe. This survey will take approximately **10-20 minutes** to complete.

If you would first like to read more about the SurPass or the European PanCareSurPass project, please visit [link].

This project has received funding from the European Union's Horizon 2020 research and innovation programme under grant agreement No 899999. The material presented and views expressed here are the responsibilities of the author(s) only. The EU Commission takes no responsibility for any use made of the information set out.

Instructions

Before you start: if you would like to delete your answer to a question, please click the gear icon on the right and select 'clear value'. For some questions we provided additional information. To make the additional information appear, please click the 'I' icon below the question.

Starting questions

1. **Please indicate your country:**

   ____

2. **Have you ever heard of the SurPass before?**
   o Yes
   o No
   o Don't know

3. **At your institution, has a previous version of the SurPass already been implemented?**
   o Yes
   o No
   o Don't know

4. **At your institution, what is the organisational unit that is responsible for the survivorship care for childhood cancer survivors?**
   o Paediatric oncology clinic/outpatient office
   o Medical Oncology[1] clinic/outpatient office
   o Survivorship care clinic
   o Other: ___________________________

5. **At your institution, is there a specific oncology-IT or a general institution-wide IT department?**
   o Specific oncology-IT department
   o Institution-wide IT department
   o Other:___________________________

Computing Infrastructure

We would like to know which of the systems below can be accessed by survivorship care staff, whether information can be downloaded, entered and/or updated and whether the systems can exchange information.

6. **Please complete the scheme below.** If **not** accessible by survivorship care staff, or your don't know, select 'no/don't know' and leave the rest of the row blank.

[1] When there is no independent survivorship care clinic and childhood cancer survivors are transitioned from paediatric oncology to medical oncology for survivorship care, please select **medical oncology clinic / outpatient office.**

	Accessible by survivorship care staff?	Name and Provider	Can information be downloaded by survivorship care staff?	Can information be entered by survivorship care staff?	Can information be updated by survivorship care staff?	Integrated transparently, via common IDs, or by other means?	API available to integrate?
National EHR	*Yes/No/Don't know*	*Free text*	*Yes/No/Don't know*	*Yes/No/Don't know*	*Yes/No/Don't know*	*Transparently/Other means/Don't know*	*Yes/No/Don't know*
Regional EHR							
Cancer Registry							
Hospital EMR							
Patient record at survivorship care clinic							
Patient record at other outpatient clinic							
Appointment Scheduling system							
Pharmacy							
Labs							
Radiology							
Clinical trial systems							
The Primary Health care (e.g., GP) Information System							
Other (e.g., database, Registry, etc.)							

7. **Which Health Data Exchange standards/interoperability frameworks are already used or currently implemented in your institution?** Please check all that apply.

○ HL7 Version 2

o HL7 Version 3
o HL7 FHIR
o HL7 CDA
o IHE (Integrating the Healthcare Enterprise) Profiles
o Other(s):_____________________
o None

8. **Please briefly explain the standards/interoperability frameworks in use.**

____

Information about childhood cancer survivors

The following questions are about which information is available to you/your institution.

9. **Please complete the scheme below.** What information about childhood cancer survivors is available to survivorship care staff? And in what form? If not available, or your don't know, select 'no/don't know' and leave the rest of the row empty. You may need to scroll right to view the entire scheme.

	Available?	In what form?	If other form, please specify:	At what level?	Can data be accessed locally and/or remotely?	By which personell?	Can data be entered locally and/or remotely?	By which personell?	Can data be updated locally and/or remotely?	By which personell?
Patient Summary / Medical History	*Yes/No/Don't know*	*Hardcopy (in paper)/Digital Word or PDF file/EMR/HL7 CDA/Other/Don't know*	*Free text*	*National/ Regional/ Hospital/ Survivorship care clinic/Don't know*	*Locally/ Remotely/ Both/ Neither/Don't know*	*Free text*	*Locally/ Remotely/ Both/ Neither/Don't know*	*Free text*	*Locally/ Remotely/ Both/ Neither/Don't know*	*Free text*
Hospital Admissions										
Diagnostic Imaging (Images)										
Diagnostic Imaging (Reports)										
Biochemical Labs										
Pathology labs										
Childhood cancer Treatment Summary										
survivorship care clinic visit report										
Survivorship passport										
Survivorship care										

appointments										
Other medical databases										

10. **Please complete the scheme below. From the available information sources, can you retrieve the following information on general medical history from the available information sources?** This information may or may not be part of the digital SurPass. If not available to you, please select 'no' and leave the rest of the row empty.

	Available?	In what form?	Free text or coded?	If coded, please specify which coding system:	Interconnected	Comment
Comorbidities	*Yes/No/Don't know*	*Hardcopy (in paper)/Electronic nformation system*	*Free text/coded*	*Free text*	*Yes/No/Don't know*	*Free text*
Allergies						
Medication (Non Cancer Related)						
Surgical Procedures (Non cancer related)						
Admissions						
Trauma						
Hereditary Syndromes						

11. **Please complete the scheme below. From the available information sources, can you retrieve the following information on cancer diagnosis from the available information sources?** This information may or may not be part of the digital SurPass. If not available to you, or you don't know, please select 'no/don't know' and leave the rest of the row empty.

	Available?	In what form?	Free text or coded?	If coded, please specify which coding system:	Interconnected	Comment
Cancer diagnosis	*Yes/No/Don't know*	*Hardcopy (in paper)/Electronicl nformation system/Don't know*	*Free text/coded/ Don't know*	*Free text*	*Yes/No/Don't know*	*Free text*

Cancer diagnosis date						
Histology-Cytology Report						
Imaging Reports						
Lab Reports						

12. **Please complete the scheme below. From the available information sources, can you retrieve the following information on cancer management from the available information sources?** This information may or may not be part of the digital SurPass. If not available to you, please select 'no' and leave the rest of the row empty.

	Available?	In what form?	Free text or coded?	If coded, please specify which coding system:	Interconnected	Comment
Surgical Intervention(s) related to cancer treatment	*Yes/No/Partly/Don't know*	*Hardcopy (in paper)/Electronic Information system*	*Free text/coded*	*Free text*	*Yes/No/Don't know*	*Free text*
Stem cell / bone marrow Transplantation(s) (Date, auto/allogeneic transplant)						
Chemotherapy (Start/End Date)						
Chemotherapy type						
Chemotherapy cumulative dose(s) (Dose/m^2)						
Chemotherapy treatment complications (need to discontinue, modify, change therapy)						
Immunotherapy (Start/ End Date)						
Immunotherapy type						
Immunotherapy cumulative Dose (Dose/m^2)						
Immunotherapy treatment complications (need to discontinue, modify or change therapy)						
Hormonal Therapy (Start /End Date)						
Hormonal therapy type of Drug(s)						
Hormonal therapy cumulative Dose (Dose/m^2)						

Hormonal therapy treatment complications (need to discontinue, modify or change therapy)						
Radiotherapy / Radionuclide therapy (Start/ End Date)						
Radiotherapy type						
Radiotherapy cumulative Dose						
Radiotherapy Site						
Radiotherapy treatment complications (need to discontinue, modify or change therapy)						

Legal aspects

The following questions are about potential legal issues related to implementation of the SurPass.

13. Please explain your dataflow/security workflow.

    ____

14. At your institution, would you be able to guarantee protection of digital SurPass (v2.0) data against unauthorised usage?
    - o Yes > Qb
    - o No > Qa
    - o Don't know > Q15

    a. Why not?

    ____

    b. How would you protect (digital) SurPass data against unauthorised usage?

    ____

15. At your institution, do / would SurPass records need to be stored for a minimum amount of time?
    - o Yes > Qa, then b
    - o No > Qb
    - o Don't know > Qb

    a. For how long do / would records need to be stored?

    ____

    b. At your institution, for how long can SurPass records be stored?

    ____

Economical aspects

The following questions are about potential economical issues related to implementation of the SurPass.

16. **To get the digital SurPass fully up and running at your IT-department, which personnel needs to be involved?**

    ____

17. **To get the digital SurPass fully up and running at your IT-department, how much time do you think you will need?** We understand this is just an estimation.

    ____

18. **Do you think you have the resources (e.g., staff, funds, knowledge) you will need to implement the digital SurPass at your IT-department?**
    o Yes
    o No > Qa
    o Don't know > Qa

    a. **What resources are / may you be lacking?** Please check all that apply.
       o Staff
       o Time
       o Funds
       o Knowledge
       o Other: _________________________

Final questions

You are nearing the end of this survey.

19. **In your opinion, what could be possible barriers (limitations) to implementing the digital SurPass (v2.0) at your IT department?**

    ____

20. **In your opinion, what could be possible facilitators (helping factors) for implementing the digital SurPass (v2.0) at your IT department?**

    ____

21. **Do you have any further comments/remarks?**

    ____

22. **Did you complete this survey by yourself, or with local language assistance from your institution?**
    o I completed the survey by myself.
    o I completed the survey with local language assistance from my institution.

Outro

Thank you so much for your time!
